# Long-term outcomes of the recovery approach in a high-security mental health setting: a 20 year follow-up study

**DOI:** 10.3389/fpsyt.2023.1111377

**Published:** 2023-05-11

**Authors:** Lindsay Thomson, Cheryl Rees

**Affiliations:** ^1^Division of Psychiatry, University of Edinburgh, Edinburgh, Scotland, United Kingdom; ^2^The State Hospital, Carstairs, United Kingdom; ^3^The Forensic Mental Health Managed Care Network, Carstairs, United Kingdom

**Keywords:** forensic mental health, recovery, longitudinal, follow up, social recovery

## Abstract

**Background:**

This study examined the outcomes of a descriptive, longitudinal cohort consisting of 241 patients initially examined in a population study at the high secure State Hospital for Scotland and Northern Ireland in 1992–93. A partial follow-up focusing on patients with schizophrenia was conducted in 2000–01, followed by a comprehensive 20 year follow-up that began in 2014.

**Aims:**

To explore what happens to patients who required high secure care during a 20 year follow-up period.

**Method:**

Previously collected data were amalgamated with newly collected information to examine the recovery journey since baseline. Various sources were employed, including patient and keyworker interviews, case note reviews, and extraction from health and national records, and Police Scotland datasets.

**Results:**

Over half of the cohort (56.0%) with available data resided outside secure services at some point during the follow-up period (mean 19.2 years), and only 12% of the cohort were unable to transition out of high secure care. The symptoms of psychosis improved, with statistically significant reductions observed in reported delusions, depression, and flattened affect. Reported sadness [according to the Montgomery–Åsberg Depression Rating Scale (MADRS)] at baseline, first, and 20 year follow-up interviews was negatively correlated with the questionnaire about the process of recovery (QPR) scores at the 20 year follow-up. However, qualitative data depicted progress and personal development. According to societal measures, there was little evidence of sustained social or functional recovery. The overall conviction rate post-baseline was 22.7%, with 7.9% violent recidivism. The cohort exhibited poor morbidity and mortality, with 36.9% of the cohort dying, primarily from natural causes (91%).

**Conclusions:**

Overall, the findings showed positive outcomes in terms of movement out of high-security settings, symptom improvement, and low levels of recidivism. Notably, this cohort experienced a high rate of deaths and poor physical morbidity, along with a lack of sustained social recovery, particularly among those who had negotiated a path through services and who were current residents in the community. Social engagement, enhanced during residence in low secure or open ward settings, diminished significantly during the transition to the community. This is likely a result of self-protective measures adopted to mitigate societal stigma and the shift from a communal environment. Subjective depressive symptoms may impact broader aspects of recovery.

## Introduction

Recovery is a key concept within the Mental Health Strategy for Scotland 2017–2027 (1). At its core, this strategy aimed to improve mental health and wellbeing services, with outcomes delivered for individuals and communities along with the expectation of recovery. More recently, forensic mental health services (FMHS) within Scotland have been subject to an independent review of service delivery (2). This review concluded that, in part due to advancements in service provision across the country, a change in person-centered practices was required to better support the rights and needs of forensic patients and their recovery.

To provide some context for FMHS in Scotland, there exists some differences in comparison to other European countries (Croatia, Italy, Portugal, and Spain) (3). In Scotland, individuals detained under the Mental Health (Care and Treatment) (Scotland) Act 2003 (4) with a history of serious violence and who pose a grave risk to the public, can be detained without any time limit. Therefore, an individual convicted of an offense can be detained within mental health services for a longer period of time than the length of imprisonment that would have been imposed for the original offense. Convicted individuals with a primary personality disorder diagnosis generally remain with the Scottish criminal justice system rather than being brought into the mental health system. In addition, there was a focus on establishing regional medium secure units and increasing the number of local low-secure units throughout the 2000s, with the three regional medium secure units opening during 2000–2013. This allowed patients to be moved from the central high secure forensic service and be located in the least restrictive security level, cared for closer to their local community and family support, and enhancing opportunities for recovery.

In Scotland, service users advocated for a relatively loose concept of recovery that was person-centered and tailored, enabling, and empowering (5). The reality of implementing person-centered recovery balances operational requirements alongside the ‘living process' of recovery. As conceived by the service user-led movement, recovery is defined as the development of social and personal recovery tailored to create a life with meaning and purpose regardless of the resolution of symptoms and the level of functional recovery attained (5). This individualistic recovery journey is supported by the principles of hope, control, and opportunity (6), continuing the belief that personal life goals can be achieved, regaining a feeling of control over their lives, and having the same opportunities as other members of society in terms of engagement and social inclusion. For the forensic patient, developing hope can center on negotiating issues of personal guilt and their offending behavior. Acquiring a sense of control over their lives is a balance between addressing public risk and supporting personal choice in advancing life goals. Fundamental to engagement and social inclusion is fostering the ability to trust (7). In general, individuals within the forensic system have experienced traumatic and disrupted childhoods (8), with personal attachment interpreted as dangerous and to be avoided (7).

As the process of recovery among users of general psychiatric services has changed over time, the concept of recovery within FMHS has also evolved.

It was relatively straightforward within general psychiatric services to move beyond the biopsychosocial model of psychiatric disorders and to adopt a person-centered approach in support of recovery. However, there were concerns regarding how such an approach and the underlying principles of hope, control, and opportunity could be applied to secure environments (7). This was in recognition of the difficulties associated with fostering empowerment, choice, and self-determination within an environment that is often considered coercive (7).

However, individuals in FMHS are subject to fundamentally the same recovery tasks as the users of general services. Individuals in FMHS need to undertake additional tasks regarding their offending behavior, with their legal status, and associated restrictions impacting upon their recovery experience (9). Balancing risk and safety with least restrictive practices and offering optimal choices can support recovery within a secure environment (7). As the central function of offender/FMHS centers upon reducing and managing risk to the public, there is a tendency for forensic outcome research to focus on low rates of recidivism and readmission as markers of success and recovery (10). There is, however, increasing focus in the literature on the broader aspects of individual recovery in FMHS (9).

In the context of Scottish FMHS and associated legislation, the Mental Health (Care and Treatment) (Scotland) Act 2003 (4), which is underpinned by the principles of the European Convention on Human Rights (11) and the Millan Report (12), rates of recidivism and readmission can be more readily interpreted as indicators of successful rehabilitation (2). These rates have been often reported in the literature when the recovery of individuals within FMHS is considered. This is likely as these data are routinely recorded, quantifiable in nature, and recorded in a relatively similar fashion across different countries, allowing for national and international comparisons (13).

Rehabilitation tasks such as compliance with appropriate medication, engagement with therapy, and skill development are the building blocks that support recovery. Recovery develops from rehabilitation; within the perspective of person-centered practice, it has been acknowledged that definitions of rehabilitation and recovery are individualistic. Recovery, however, is generally interpreted as enabling individuals to “pursue their own unique life goals in the presence or absence of continuing symptoms” (7). Consideration must therefore be given to the nature of what is being explored: rehabilitation or recovery. For the purposes of this study, and in embracing the individualistic nature of rehabilitation and recovery and their interdependence, we have interpreted both rehabilitation and recovery under the broad umbrella term “recovery”.

The main objective of this study was to examine the recovery process of a group of patients over time, utilizing a descriptive longitudinal design. This cohort was initially identified as part of a whole population survey that had been conducted in 1992-3 (14) and subsequently revisited in 2000-01 (15). The baseline study was based on the biopsychosocial recovery model, and the captured variables reflect this perspective. However, a new element of the study was added, focusing on recovery and adopting a person-centered approach that emphasized hope, empowerment, healing, and connection (16).

In embracing the fact that recovery for individuals in FMHS involves additional offender or legal-related tasks, recovery was conceptualized in broad terms to examine all aspects of the movement from high secure care toward the community. For this study, clinical, functional, social, and personal recovery, as outlined by Drennan and Alred (17), were examined. In summary, clinical recovery centers upon symptom resolution, with functional recovery focusing on skills training and developing capabilities for undertaking life tasks (including employment and relationships). Social recovery relates to social inclusion/exclusion and the impact of stigma upon an individual, with personal recovery focusing on the concepts of hope, control, identity, and meaning in life (17). Finally, offender recovery has been interpreted more in terms of recidivism than delving into identity issues surrounding an individual's offense.

Due to the historical grounding of the study, it was acknowledged that some elements may be more embedded within the concept of rehabilitation than recovery. The study provided information to evaluate and better target interventions to support FMHS users across the security levels in their recovery journey.

The research question was as follows:

1. What happened to patients who required high secure care in 1992/93 over a 20-plus year follow-up period?

## Methods

Sample baseline characteristics and the methodology adopted, including the process for tracing the previous participants, data collection tools, and data extraction from health and national data sets, were comprehensively described in previous studies and will only be summarized in this study (18, 19).

### Sample

The State Hospital Survey (SHS) described a whole population cohort of 241 patients, with 88.4% being men with a mean age of 35.8 years, and 11.6% being women, with a mean age of 32.4 years, who were detained within the high secure State Hospital, Carstairs, Scotland, at some point between 25th August 1992 and 13th August 1993 (14). Table 1 provides an overview of baseline characteristics and participation at follow-up. At baseline, 70% of the cohort received a primary diagnosis of schizophrenia and 5.4% antisocial personality disorder (ASPD), with comorbid ASPD diagnosed in 28.9%. Individuals were subject to case note review and clinical interviews wherever possible. During 2000-01, a partial follow-up of 169 cohort members with a baseline (SHS) diagnosis of schizophrenia was undertaken (15). The 20 year follow-up was initiated in 2014, with a year-on-year case note review undertaken for the mean of 19.2 years (Baseline to 31st December 2014 or date of death). Mortality data were collected for a mean of 21.1 years. Cohort characteristics were extensively described in a previous study (14) and reflected a relatively young but severely ill population who experienced significant childhood adversities and reported higher than average rates of chronic physical ill health.

**Table 1:** Sample overview at baseline and 20 year follow-up.

**The State Hospital survey (baseline)**		***N* (%)**
Sex	Men	213 (88.4)
	Women	28 (11.6)
	**Total**	**241 (100)**
Mean age	Men	35.8 (17.5–67.1) years
	Women	32.4 (17.2–60.3) years
Primary diagnosis	Schizophrenia	169 (70)
	Antisocial personality disorder (ASPD)	13 (5.4)
	Comorbid ASPD	66 (28.9)
Inpatient history	Mean lifetime stay to baseline (1992/93)	9.3 years
**20 year follow-up**		***N*** **(%)**
	Deceased (to 31st December 2017)	89^*^ (36.9)
Gatekeeper decision	Approved for invite to study	87 (36.1)
	No capacity to consent/too unwell	37 (15.4)
	Resident outside Scotland (no gatekeeper info)	21 (8.7)
	No appropriate gatekeeper (within Scotland)	4 (1.7)
	Lost to study	4 (1.7)
	**Total**	**242** ^*^ **(100)**
	**Overall participation rate**	**66/87 (75.9)**
Consent provided	Interview + case note review	48/66 (72.3)
	Case note review only	18/66 (27.3)
	Police Scotland criminal history summary	59/66 (89.4)

^*^One person double counted as they died following participation.

Bold was used to highlight the total values (cohort and participation rate).

At the 20 year follow-up, of the 241 cohort, 36.9% were noted as deceased^1^, which was at the close of the mortality figures taken on 31 December 2017, and gatekeepers approved 36.1% for invitation to study and 15.4% as either not having the capacity to consent or being too unwell mentally or physically to be included in the study. Almost 9% of previous participants were residents in other regions of the UK (RUK) or overseas. Despite considerable efforts, we could not access relevant information to secure RUK gatekeeper decisions. A small number of individuals (1.7%) were either lost to study or were removed from mental health and general practitioner services, and thus, we could not locate an appropriate gatekeeper (1.7%).

Of those who were approved by gatekeepers for approach and invited to participate, 75.9% consented to the study in some way (interview and/or case note review). Of those who consented, 72.3% agreed to be interviewed. In addition, 89.4% consented to Police Scotland, providing a summary of their criminal history from the baseline to December 2014. Permissions were obtained for case note information and Police Scotland data to be sourced for deceased cohort members.

### Ethics

The authors assert that all procedures contributing to this study comply with the ethical standards of the relevant national and institutional committees on human experimentation and with the Helsinki Declaration of 1975, as revised in 2008. The study was approved by South East Scotland Regional Ethical Committee 01, reference 15/SS/0015. Supplemental approvals and the wider study protocol are detailed in a previous publication (18). Written informed consent was obtained from all living participants following a face-to-face introduction to the study by the researcher. Individuals had the opportunity to consider the information and the extent to which they wished to participate, and they held the right to withdraw at any point. The data of deceased individuals were also included in the follow-up, with a range of NHS ethical and other permissions secured to access and review central and local health records (18). Access to deceased records was sought due to the high level of mortality among those experiencing severe mental illness as reported in the literature. Ethical permission was also secured to obtain data from keyworkers using the World Health Organization Disability Assessment Schedule (20), regardless of the participation decision of the cohort members.

### Procedures

After tracing the cohort members and their current care team using data provided by National Services Scotland (NSS) and the National Health Services Central Register (NHSCR), a gatekeeper approach was implemented (18).

Consultant psychiatrists (responsible medical officers, RMO) determined whether the previous SHS participant was allocated to their service, possessed the capacity to consent, and was physically and/or mentally well enough to be invited to participate in the study. Only after receiving approval from the RMO or other suitable gatekeepers (e.g., general practitioners) was an invitation to participate extended.

Considering the vulnerable participant group and research topic, the study was specifically designed to offer maximum flexibility and encourage participation. To build trust and rapport with potential participants, a single researcher conducted the study introduction, sought informed consent, and collected all data. The participants could consent to all or some of the following:

- Participant quantitative and/or qualitative interview (with optional consent for audio recording),
- Case note review and/or,
- Summary of criminal history since baseline.

Baseline variables including; demographics, admission history and characteristics, medication, self-harm and abuse history, childhood development and family, mental health history, physical health, forensic history, drug and alcohol use, and functional ability within the ward environment were compared between those contributed data to the follow-up and those who were too unwell or lacked the capacity to consent, refused to participate, or were lost to the study cohort. Individuals from RUK and overseas were excluded from the analysis. Significant differences were observed in terms of age at baseline, the time in current high-security admission prior to baseline, and the total length of time as a psychiatric inpatient. The group that provided data for the study demonstrated higher median scores.

### Data collection

The 20 year follow-up amalgamated, where possible, relevant case notes and interview data that had been previously gathered (14, 15) with new follow-up information. Data were collected by using previously used tools through interviews and/or case note reviews and, where possible, keyworker/staff interviews (18). Participant interviews were conducted at baseline, 10-year follow-up, and 20 year follow-up. Appendices A–E, located within the supplementary information, detail the tools applied at each study time period, the case note review, quantitative and qualitative interview schedules, and how each tool was utilized within the field (18).

For participants who had provided consent, a case note review was conducted every year from baseline, or from 2002 if they were included in partial follow-up, to obtain an overview of the individual's clinical and functional recovery to December 2014. For participants who died, the same was conducted until December 2014 or until date of death. The review was conducted only if participant case notes were still available.

The 20 year follow-up was conducted in the context of recovery and included, in addition to the previously used suite of interview tools, the original 22-item questionnaire about the process of recovery (QPR) (21) and a semi-structured interview of approximately 1 h duration, which was based around seven elements of recovery: hope, a secure base, a sense of self, supportive relationships, empowerment and inclusion, coping strategies, and a life with meaning and purpose. The interview schedule can be viewed in Appendix E in the Supplementary material.

These tools allowed for the exploration of social and personal recovery. In the absence of data relating to forensic cohorts and the performance of the QPR, the decision was made to use the original 22-item tool, as this would also allow assessment of the revised 15-item measure (22). Reflexive thematic analysis (23) was applied to the qualitative data using an inductive, constructionist method, and interpretation was conducted through an informed, non-clinical lens.

To complement other data collection efforts, permission was obtained to extract data from National Services Scotland (NSS) data sets (Scottish Morbidity Records SMR 04 Psychiatric Inpatient and SMR00 Outpatient-Psychiatric Only) to accurately establish the journey of consented/deceased individuals through services and to establish the location of case notes for review. Data were also extracted for deceased/consented individuals in relation to general hospital inpatient and day patient (SMR 01) events to more robustly examine physical health (18, 19). Criminal conviction data supplied by Police Scotland provided a robust method of assessing offender recovery.

An overview of the main findings by recovery category is presented.

## Results

### Clinical recovery

**Figure 1:**
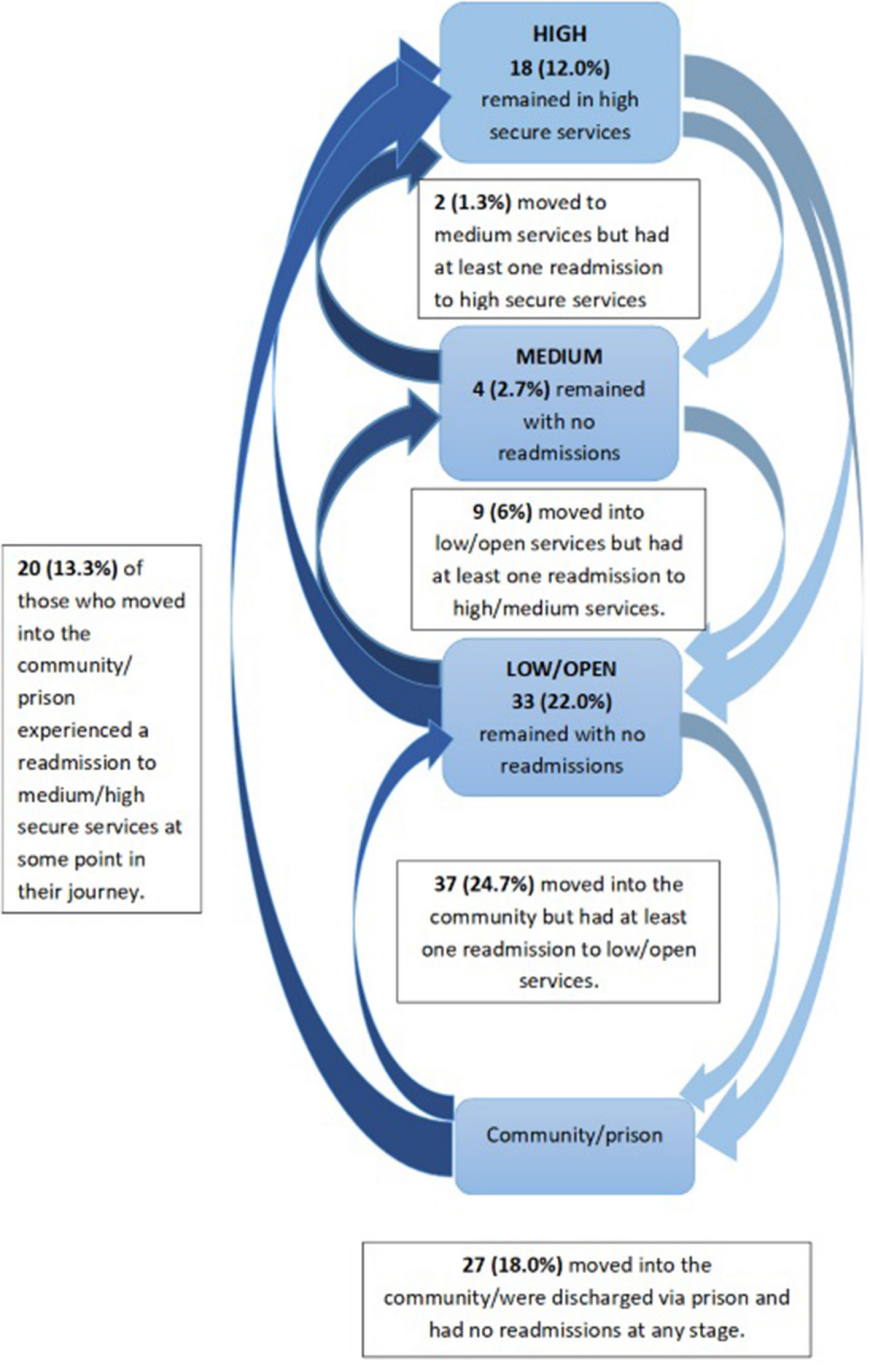
Lowest level of security achieved and readmissions.

The journey through services was explored to provide an overview of clinical recovery. Data from NSS SMR04 (mental health inpatient/day patient) describing the full location journey from baseline (1992/93) to 31st December 2014 or the date of death, were available for 62.2% of cohort members. Figure 1 examines the lowest security level achieved and any readmissions experienced. After the baseline survey, 12.0% of the cohorts did not move on from high secure services. For 4.0% of individuals, medium secure services was the lowest level attained; 28.0% transitioned to low/open services; and 56.0% experienced at least some time in the community/possibly prison. Robust data were not available on detention in prison except where individuals were discharged to prison. Figure 1 outlines patient movement and highlights the number of patients who required readmission to higher levels of security.

It should be noted that the first Scottish medium secure service opened in 2002, with the other two centers opening in 2007 and 2014. A significant difference was observed between baseline legal status and lowest security level achieved [X^2^(6) = 22.7, *p* = 0.001, Cramer's V = 0.28]. Of those achieving the lowest security level, either high or medium secure care, 83.3% were subject to criminal procedures at baseline, where the lowest level attained was low/open secure services, 73.8% were criminally detained. Of those under civil detention at baseline, 42.4% were now residents in the community with occasional readmission. Similarly, 73.9% of those who had been under prison transfer for treatment orders resided in the community and experienced occasional admissions.

Using NSS SMR04 data, the mean number of admissions to each security level and the average days spent at each level from baseline (1992/93) to 31 December 2014 or the date of death were established and can be viewed in Table 2. After the baseline (SHS) study, the mean high-security admission was 6.8 years (median 4.3 years, range: 0.09–22.4). Individuals subject to restrictions on discharge, excluding prison transfers (n = 81), had a mean stay of 9.4 years (median 6.1, range: 0.15–22.4) in high security. Non-restricted individuals and prison transfers (n = 160) spent a mean of 5.4 years (median 3.5, range: 0.09–22.4) in high security. The maximum time in the study was 8,187 days or 22.4 years. Mortality analysis was conducted for a mean of 21.1 years (median 25.1, range: 0.61–25.4). The mean admissions were 4.2 (median 3.0, range 0–38), with admission defined as a change in security level (i.e., high to medium security) with baseline admission counted as 1.

**Table 2:** Admissions, discharges and length of stay from baseline until 31.12.2014 or the date of death (State Hospital and SMR04 data).

**Admissions, discharges and length of stay**	**Number of cases providing data**	**Mean**	**Median**	**Minimum**	**Maximum**
^*^High secure inpatient admissions	*n* = 241	1.3	1	1	7
Total number of days spent in high secure care	*n =* 241	2,469.9	1,561	33	8,187
Medium secure inpatient admissions	*n =* 163	0.2	0	0	2
Total number of days spent in medium secure care	*n =* 153	253.9	0	0	7,219
Low secure/open ward admissions	*n =* 158	2.7	1	0	36
Total number of days in low secure /open wards	*n =* 152	2,191.3	1,442	0	8,002
Total inpatient admissions	*n =* 150	4.2	3	0	38
Total days as an inpatient, any security level	*n =* 150	5,137.5	5,720	130	8,187
Total non-high secure inpatient days	*n =* 150	2,437.7	1,755	0	8,002
Total discharges to community/prison setting	*n =* 153	2.4	1	0	35
Total days within community/prison setting	*n =* 152	1,754.1	733	0	7,711

^*^Baseline admission counted as 1.

The change in symptom profile over time was assessed using the Manchester Rating Scale (MRS) (24), which provides an overview of positive, negative, and affective symptoms. The MRS was delivered at the 20 year follow-up to those consenting to interview. Table 3 presents the findings of the chi-squared test over time, with statistically significant differences noted between baseline and the 20 year follow-up interview in relation to flattened affect [X^2^(1) = 8.9, *p* = 0.003, Cramer's V = 0.22], coherently expressed delusions [X^2^(1) = 19.1, *p* < 0.001, Cramer's V = 0.32], depression [X^2^(1) = 8.5, *p* = 0.004, Cramer's V = 0.21], and poverty of speech [X^2^(1) = 13.9, *p* < 0.001, Cramer's V = 0.27]. No statistically significant differences were noted with regard to incoherence and irrelevance of speech, anxiety, hallucinations, and motor retardation.

**Table 3:** Clinical symptoms as defined by the Manchester Rating Scale (24) over time (baseline/20 year follow-up).

**Manchester rating scale item**	**Baseline interviews** ***N*** = **142**		**20 year follow-up interviews** ***N*** = **48**		Significance^*^
	***n***	**Percentage**	***n***	**Percentage**	
Depression	49	34.5	6	12.5	0.004
Anxious	59	20.4	7	14.6	ns
Flattened incongruous affect	50	35.2	6	12.5	0.003
Psychomotor retardation	9	6.3	7	14.6	ns
Coherently expressed delusions	61	43.0	4	8.3	< 0.001
Hallucinations	33	23.2	5	10.4	ns
Incoherence and irrelevance of speech	16	11.3	3	6.3	ns
Poverty of speech	3	2.1	8	16.7	< 0.001

^*^Chi square, Asymptotic significance (2 sided), Fisher's exact test used when appropriate. ns, non significant.

### Physical health

The physical health of the cohort was primarily assessed through the examination of death certificate information and general hospital inpatient/day patient event (SMR 01, baseline to 31 December 2014) data centrally held by NSS for both deceased and consented individuals. Data have been comprehensively examined in a previous study (19). In summary, at the close of mortality figures (31 December 2017), 36.9% of the individuals were deceased. At the point of death, 51.7% of individuals were living in the community, 30.3% resided in low/open wards, 15.7% were within high security, and 2.2% were in prison.

The mean age at death was 55.6 years (range 30.5–84.7), with 77 (36.2%) of the men dying at a mean age of 56.6 years (range 30.5–84.7) and 12 (42.9%) women at a mean age of 48.9 years (36.2–66.1). Years of birth for the deceased cohort were applied to the expectation of life by gender and selected age, Scotland, 1861 to 2017 table (25). Deaths were categorized as premature when the participants died before the predicted life expectancy based on year of birth or closest period. Premature death was experienced by 67.5% of men and 91.7% of women. The average number of years of potential life lost was 14.9 years (0.09–35.7) for the men and 24.1 years (range 5.2–35.8) for the women.

Premature death was primarily the result of respiratory disease/cancer (31.7%), while circulatory disease/event accounted for 19% of deaths. No significant differences by cause were noted between premature and post-expected-age deaths. Suicide accounted for 5.6% of overall deaths, accidental deaths for 3.4%, and drug- and alcohol-related deaths for 4.5%.

Examining morbidity, we found that there were no significant differences in mean endocrine, nutritional and metabolic disease diagnoses, or diseases of the circulatory system between those who died prematurely and those who remained alive. However, those dying prematurely demonstrated significantly higher mean respiratory disease diagnoses (1.44 diagnoses, *p* = 0.002) compared to living participants. Similarly, significant differences were apparent between prematurely deceased (4.05 diagnoses, *p* = 0.005) and living participants when the means of injury, poisoning, and other external causes of injury were examined (1.31 diagnoses). It was not possible to distinguish incidents of self-harm from accidental events.

### Functional recovery

Dysfunction was assessed through a keyworker interview with the WHODAS (20). Data were obtained in relation to 94.6% of cohort members at baseline and 59.1% living Scottish-based cohort members at the 20 year follow-up. Higher scores represent greater dysfunction.

Analysis with Mann–Whitney U test, demonstrated a significant (two-tailed) difference in overall dysfunction at baseline between those with high or medium secure care as their lowest level of security (*n* = 23 Mdn = 0.75) and those who attained the community as their lowest level (*n* = 76, Mdn = 0.13), U = 640.0, z = −2.03, *p* = 0.042, r = −0.20 and in disturbed ward behavior; high or medium (Mdn = 5.00), community (Mdn = 3.50), U = 628.5, z = −2.05, *p* = 0.40, r = −0.20. However, when considering contact with the outside world, those who progressed to the community (Mdn = 0.67) exhibited significantly higher dysfunction at baseline than those remaining in high or medium security (Mdn = 0.33), U = 633.5, z = −2.05, *p* = 0.039, r = −0.20. There was no significant difference between groups at baseline in relation to the nurses's opinion of dysfunction or functioning within occupational services. No significant differences in baseline functioning were observed between individuals with high or medium security and those with low or open security as their lowest attained level of security.

Functional change over time was examined between the baseline and the 20 year follow-up. The Wilcoxon signed-rank test indicated significant differences in dysfunction scores in terms of nurses' opinion of dysfunction (z = −2.20, *p* = 0.028, r = −0.59) and contact with the outside world (z = −2.53, *p* = 0.11, r = −0.68) for individuals within high- or medium-secure (*n* = 14) services, there was an increase in median scores over time, from Mdn 0.43 to 0.77 and Mdn 0.33 to 0.83, respectively. For those with the lowest security level or low/open (*n* = 17) security, a significant difference in disturbed ward behavior was observed (z = −2.12, *p* = 0.34, r = −0.51), with a decrease over time observed (Mdn 4.00 to 1.00). There was no significant difference observed between the baseline and the 20 year follow-up in overall dysfunction for individuals progressing into the community as their lowest level of security.

In line with Drennan and Alred's (17) recovery definitions, social recovery focuses on social inclusion/exclusion and the impact of stigma on an individual. Functional recovery has been conceptualized as linked to skills training and the development of capabilities for undertaking life tasks. Maintaining a partnership and securing employment are considered functional recovery aspects. The overlap between functional and social recovery is, however, recognized. At baseline, 85.5% described themselves as single, with 4.1% married or cohabiting and 10.4% divorced or separated. At 20 years, follow-up death certificate information was examined to obtain robust relationship data. Of the 89 deaths, 74.2% were recorded as single, with 6.7% married or widowed, 7.9% divorced, and 11.2% with their status unknown. Keyworkers of 24 individuals resident in the community at the time of the invitation to study indicated that 8.3% had a partner and 4.2% had childcare responsibilities.

At baseline, 23.2% of the participants reported ever being employed; however, at the 20 year follow-up, no participants were in paid employment, with 11.1% in some form of supported work style placement.

### Offender recovery

Data were extracted from the Police Scotland Criminal History System (CHS) for 59 participants and 92 deceased individuals (three died after the official close of mortality figures); some convictions were removed in accordance with Police Scotland's Weeding and Retention policy for CHS (26), and 10.6% of the participants declined to consent to search police data.

From 1993 to 31st December 2014, 66.1% of participants and 32.6% of deceased individuals had no convictions recorded. Over 15% of participants could not be traced, and these were mainly long-term inpatients. Over half of the deceased individuals (58.7%) were untraceable. The untraceable individuals were anticipated as confirmed deceased individuals were removed from police data sets. Convictions were returned for 21.1% of traceable deceased and 24.0% of traceable participants. An overall conviction rate of 22.7% was observed, with violent offenses committed by 7.9% of individuals with convictions. Together, these 20 individuals committed 54 crimes. Table 4 details the security level at the time of the offense and Table 5 details the types of offenses committed according to Scottish Government Justice Department (SGJD) groupings (27).

**Table 4:** Security levels at the date of the offense.

**SGJD group**	**Scottish Government Justice Directorate (SGJD) Crime codes/Classifications (Groups)**	**Location** ***N*** **(%)**			
		**Community**	**Open**	**Low**	**Medium**
1	Crimes of violence etc.	5 (31.3)	2 (11.7)	5 (31.3)	5 (31.3)
2	Sexual offenses	3 (75.0)	.	1 (25.0)	.
3	Crimes of dishonesty	6 (100)	.	.	.
4	Fire raising, malicious mischief etc.	1 (100)			
5	Other crimes	18 (90.0)	.	2 (10.0)	.
6	Miscellaneous offenses	5 (83.3)	.	1 (16.7)	.

**Table 5:** Offenses described by Scottish Government Justice Department (SGJD) groupings.

**SJDG group**	**Scottish Government Justice Directorate (SGJD) Crime codes/Classifications (Groups)**	**Total *n* (%)**	**Subgroup**	***n* (%)**
1	Crimes of violence etc.	17 (31.5)	Serious assault	5 (29.4)
			Robbery and assault with intent to rob	2 (11.8)
			Other	10 (58.8)
2	Sexual offenses	4 (7.4)	Adult victim	3 (75.0)
			Child victim	1 (25.0)
3	Crimes of dishonesty	6 (11.1)	Housebreaking	3 (50.0)
			Opening lockfast places	1 (16.7)
			Theft	1 (16.7)
			Other	1 (16.7)
4	Fire raising, malicious mischief etc.	1 (1.8)	Fire raising	1 (100)
5	Other crimes	20 (37.0)	Crimes against public order	12 (60.0)
			Crimes against public justice (court)	4 (20.0)
			Offensive weapons	2 (10.0)
			Drugs	2 (10.0)
6	Miscellaneous offenses	6 (11.1)	Other^*^	6 (100)
	Total	54 (100)		

^*^Includes for example; Prison (Scotland) Act 1989 (not elsewhere classified), social work and community services offenses, Child Support Act 1991 etc.

The case note review supported the Police Scotland data and confirmed that no homicides, attempted homicides, or acts of rape were committed. The sexual assaults were also examined through case-note review, with all convictions relating to non-contact sexual offenses committed by three individuals.

### Personal recovery - quantitative

The Mann–Whitney *U* analysis of the Questionnaire about the Process of Recovery (QPR) data captured through an interview at the 20 year follow-up indicates a statistically significant difference in self-reported ratings of recovery in terms of the 22-item intra-personal scale (U = 33.5, Z = −2.14, *p* = 0.32, r = −0.43) and the revised 15-item measure (U = 34, Z = −2.11, *p* = 0.034, r = −0.43) between the high or medium (median 43.4 and 38.4 respectively) and low/open secure environments (54.4 and 47.9, respectively). No other significant differences were observed in terms of security level at the 20 year follow-up interview. QPR data were explored using the Mann–Whitney *U* test and Kendall's tau in relation to a range of baseline measures examining demographic, diagnostic and symptomatic, forensic/legal, functional, and behavioral variables, with no significant differences observed. The correlation with Kendall's tau demonstrated that depression at baseline (1992/93) as rated within the Manchester Rating Scale, which was delivered at the interview, and 15-item QPR scores were significantly associated (τ = −0.28, *p* = 0.017, *n* = 46). To further explore this finding using Spearman's correlation coefficient (rho), 15-item QPR scores were assessed against depression as reported at baseline (*n* = 46) using the MADRS, which was also delivered at the interview. A significant (two-tailed) negative correlation was observed for reported sadness (subjective) (r_s_ = −0.34, *p* = 0.021). In examining MADRS scores at first follow-up (2000/01) among those with a schizophrenia diagnosis (*n* = 23) against 15 item QPR scores, significant negative correlations were observed for reported sadness (subjective) (r_s_ = −0.49, *p* = 0.018), pessimistic thoughts (r_s_ = −0.44, *p* = 0.035), and suicidal thoughts (r_s_ = −0.46, *p* = 0.026). Significant negative correlations were also observed between 20 years follow-up (*n* = 46) MADRS and 15 item-QPR scores; apparent sadness (r_s_ = −0.45, *p* = 0.002), reported sadness (subjective) (r_s_ = −0.58, *p* < 0.001), inner tension (r_s_ = −0.41, *p* = 0.005), concentration difficulties (r_s_ = −0.31, *p* = 0.036), inability to feel (r_s_ = −0.44, *p* = 0.002), pessimistic thoughts (r_s_ = −0.32, *p* = 0.033), and suicidal thoughts (r_s_ = −0.46, *p* = 0.001).

### Personal recovery - qualitative

To examine the subjective recovery experience of transitioning from high secure services to the community, *N* = 10 qualitative interviews were conducted with individuals who were residents within the community at 20 year follow-up. At baseline, 80% of the group had received a primary diagnosis of schizophrenia. By the time of follow-up, half the cohort were living in supported accommodation or receiving a comprehensive support package within their own homes. Eighty of the interviewed cohort were men. Reflexive thematic analysis (23) was used to generate four global themes, which are detailed in Table 6 and which apply to both secure FMHS and community environments. These themes include working on the foundations, seeing change, developing and practicing the skills to move forward, and the framework of recovery. The informant quotes within each global theme were structured by organizing themes/domains.

**Table 6:** Personal recovery organizing and global themes.

**Personal recovery**	
**Organizing themes/domains**	**Global themes**
	Working on the foundations
Hope	
Barriers to recovery	
Treatment plan	
	Seeing change
The a'ha moment	
Changing identities	
	Developing and practicing the skills to move forward
Appropriate coping mechanisms	
Insight	
Sense of progression	
Personal and institutional challenges	
Staff feedback	
	The framework of recovery
“Recovery”	
Self-protective measures	

#### Working on the foundations

“*Hope”* is presented as hopelessness at their situation when individuals reflected on their experience of being detained within a high secure FMHS:

“*I've come a hell of a long way, when I was in Carstairs [high secure care], I never thought I was getting out.” [136]*

Hope and confidence continued as a concept in the community, predominantly among those who did not feel particularly confident about their recovery.

“*I have been thinking ‘can I really do this?', and the answer is ‘yes, I can do it.” [046]*

A myriad of potential “*barriers to recovery”* exerted their strongest influence after admission and detention within high secure FMHS and at points throughout the pathways that individuals negotiated through secure FMHS. There was a strong recognition of immature and inappropriate behaviors displayed while in the high secure FMHS.

“*Thought [I could] pull the wool over people's eyes, taking drugs, this, that and the next thing bending every rule you could imagine.” [154]*

“*You know, I was being very offensive…” [154]*

Although certain barriers, such as addiction issues and media interest on admission, may diminish over time, they can become relevant and potentially influential again at any point, particularly when reintegrating into the community.

Through “*treatment plan”* development, the main elements supporting recovery were established: medication, psychological therapies, and life skills. The informants were clear about the importance of medication and finding the correct medication.

“*Well, I got medication, I hadn't been on medication, that helped, that started getting me out of the fantasy because I had been living in my own wee world…” [047]*

“*You are put on the medication and whatnot, and sometimes, like for some guys like myself, it takes a long time for the medicine to work because, at that point, I wasn't interested in medicine, [pause] I thought, [pause] I know better than the doctors.” [154]*

The influence of psychological therapies was also noted.

“*It was like cognitive skills, an understanding where other, other peoples coming from, and everybody got the right to an opinion. [pause] There was role play involved, and eventually me and a lot of the other guys that went there, that didn't like it at first, we used to look forward to going, for the role play and that, you know it was really cool.” [154]*

“*I went on the R&R [reasoning and rehabilitation] course*, ***and that totally got a grip of*** ***me that.”*** *[154]*

#### Seeing change

Some individuals reported experiencing what we have termed the “*A'ha moment”*: the moment when the path to your goal becomes obvious.

“*I was sitting watching big Tam and somebody else one day, and* ***that's when it hit me*, ***and I thought ‘****that's how the staff and the*** ***doctors must see us!***”*' [154]*

“*I honestly, I don't know what happened, but I just I just got like a big [individual making a wooshing sound], you know, right in the face one day, and I thought “****What am I doing*,** ***what am I doing here****?”' [154]*

Another factor at this stage is the negotiation of “*changing identities”*. In addition to negotiating their new identity as a forensic mental health patient detained within high secure care who has demonstrated seriously aggressive behavior, including violence and aggression, individuals also need to manage other changing identities and the perception conveyed by doctors.

“*Classed insane by 5 doctors, I remember it being said, and it wasn't a nice feeling, let me tell you.” [183]*

“*A doctor told me… that I am a very nervous person, insecure person, my nervous ability, the way I walk and stuff, it's all the package.” [183]*

“*I was told by a doctor in the State that I would never be the person that I was before I turned insane.” [136]*

Individuals were also able to self-reflect upon the person they had viewed themselves as when detained in high secure FMHS:

“*I see myself in the State [hospital] and prison as a stupid wee boy who thought he was clever.” [154]*

“*I won't go into detail, but I was a risk to others.” [047]*

They were able to contrast that with the person that they saw themselves as then and 20 years after that experience:

“*if I am looking at myself now, I see a much more mature person, an adult, grown up.” [154]*

“*I have decided to make the changes, and I have accepted that I have to change, [pause] get better, to survive, to achieve what I want to achieve.” [046]*

#### Developing and practicing the skills to move forward

Central to the question of being able to move on from high secure FMHS to a lower level of security and eventually the community was the development of “*appropriate coping mechanisms”* and “*insight”*.

“*I was challenging myself to get better, bit by bit. I was doing things I didn't want to do, forcing myself into groups, I didn't want to do it, [pause] I was eating things I didn't want to eat [pause] this challenge in my head was to get better.” [183]*

“*I was sitting in my room one day and I was thinking, I'm here, what am I doing here, pushing against the system, and I am still here I might try it the other way.” [154]*

This goes hand in hand with the development of a “*sense of progression”*, which can be fuelled not only by improvements in insight and recognizing changes within themselves since admission but also by the physical transition from a higher to a lower level of secure FMHS care. “*Staff feedback”*, formal and informal, supported progression.

“*When people are telling you … it gives you a bit of self-praise as if to say, “well I am doing something right, I am moving on” but people are noticing, it's noticed, it's getting recognized.” [046]*

“*She [the doctor] basically said that in* ***my report*** *[participant name] is the most model patient in this ward, and she said that's saying something!” [154]*

As individuals' coping mechanisms and level of insight improve, they become better equipped to handle “*personal and institutional challenges”* and advance their recovery. These challenges may manifest in various ways, such as being separated from family during times of grief or encountering problems when attempting to secure discharge pathways and appropriate resources to facilitate the transition to the community or lower security settings. How individuals choose to address these challenges can greatly impact their recovery, either by promoting progress or triggering a downward spiral.

“*I looked down at this nip [whisky], and I thought, that man, Dr* ^*^, has just put his whole trust in me to go to my mum's funeral to come back to the wake, and if I drink that, then, that's me betraying his trust straight away.” [154]

“*They were blocking my release, and I was taking it personally, [pause] I don't know, I don't know how I kept going.” [047]*

#### The framework of recovery

For most individuals, the concept of “*recovery”* was associated with living in the community, which is often the goal throughout life in a secure FMHS: being independent and getting back to life in the community. There was a recognition that their illness needs to be monitored and managed by them to prevent a return to services. They have to practice appropriate coping mechanisms to promote health and seek help if it is too much for them to handle. Having their own home and space is a key concept, along with running their own day-to-day lives.

“*[previously] I wouldn't even have dreamt or thought about this or having a flat on my own. I thought I was going to be in [secure FMHS] care all my life”. [136]*

“*[Recovery means] Understanding what you've been through, what has it all been about.” [079]*

“*Making your own decisions, getting better mentally and moving on in life. Back when I was in Carstairs [high secure FMHS], I couldn't make my own decisions, but now I can, I feel much stronger now, and I can make my own decisions now.” [046]*

Although living in the community is often viewed by forensic patients as the pinnacle of recovery, it comes with the realization that the “ideal” life is harder to attain than first thought. Individuals voiced some concerns regarding leaving themselves emotionally vulnerable and took “*self-protective measures”*.

“*I could meet someone now and chat away to them but, for to become friends, I don't really commit myself that far.” [154]*

“*In the community, I know a lot of people, they don't even know I was sick in the State [high secure FMHS].” [183]*

This also extended to potential harm from others finding out their index offense or even that they had been in The State Hospital.

“*I don't tell lies about myself. I sometimes miss out things that perhaps I should mention. I pick and choose rather what to say”. [079]*

“*I speak to people, and I don't tell anyone what happened in the past, it's a long time ago”. [057]*

Self-protection and, to a certain extent, isolation came across as the price that had to be paid for their index offense. This was made all the more difficult because they no longer recognized the person who had behaved in such a way that admission to high secure FMHS care was required.

### Social recovery

Ten qualitative interviews, conducted with individuals who were residents in high and medium secure FMHS at 20 year follow-up, were analyzed using reflexive thematic analysis (23) and interpreted through the lens of social recovery. As outlined in Table 7, one global theme, “*negotiating interpersonal relations”*, was generated. The theme was underpinned by five organizing themes/domains: friendships, family, intimate relationships, openness, and community. To explore how the global theme evolved under the influence of reduced restrictions and physical security, interviews with individuals from the baseline cohort who had progressed beyond high or medium secure care were examined. Ten interviews with individuals residing in low/open FMHS and 10 living in the community were analyzed from the perspective of the five organizing themes/domains.

**Table 7:** Social recovery organizing and global themes.

**Social recovery**	
**Organizing themes/domains**	**Global themes**
	Negotiating interpersonal relations
Friendships	
Family	
Intimate relationships	
Openness	
Community	

#### Friendships

Interviews with individuals who remained within high or medium secure FMHS at 20 years follow-up demonstrated that friendships appeared mostly superficial;

“*It's more like fair-weather friends in here…” [high/medium 127]*

Staff were not mentioned in terms of friendship or even positive relationships. Individuals readmitted from the community spoke of outside friendship groups without mentioning their surrounding peers. In contrast, individuals resident in low-security FMHS or open wards at the 20 year follow-up spoke of staff, both past and present, in very warm, genuine tones, with peer friendships and their importance more readily acknowledged:

“*I have a close friend in here, and he knows everything there is to know about me and I talk to him about a lot of things.” [low 003]*

Among individuals now resident in the community at 20 year follow-up, there appeared to be a reversion to superficial friendships or acquaintances;

“*I know people from college, this that and the next thing but I don't, you know, invite them back to my flat or anything.” [Community 154]*

Community residents appeared to adopt a protectionist attitude, with a need to keep themselves distanced from others outside the hospital/mental health community.

#### Family

Family visits and contact were far more important than making friends for individual residents within high or medium secure FMHS. Lists of the family members who came to visit were readily rhymed off, with such visits keenly anticipated. Family visits provided contact with the outside world and an alternative, important opinion about care options. Being able to state that family came to visit tended to enhance their self-image.

“*My brother and his wife, when they come up and visit me, give me their support and things…[it's] a little boost.” [High/ medium 159]*

The family remains important to individuals resident in low-security FMHS or open wards, with this person explaining why he considers family important to recovery;

“*I think if you have a bigger family, they inspire you to move on and help you. Some of these boys don't have any family at all, and all they have is a nurse and a social worker. They don't have the will to move on if they don't have a family.” [low 003]*

This outlook could explain why staff friendships become more important as family ties loosen over time with the deaths of parents and siblings. Among those living in the community, the family remained important, but there was occasionally a sense of not wanting to bother them;

“*My nephew, I am quite close to, that's in his nature, but he's a married man, he's a granddad now but even at that, you don't want to mess with his life.” [Community 183]*

#### Intimate relationships

The notion of intimate relationships was considered part of the internal goal setting by residents in a high or medium secure FMHS. This included consideration of how to navigate the issue of sharing their journey through forensic mental health services:

“*I'd rather be open from day one. When is the right time to tell someone about your past, at the start or 18 months down the line when you've got issues of trust?” [high/medium 064]*

While living in higher levels of secure FMHS care, the negotiation of a relationship is generally theoretical, those resident within low-security FMHS or open wards are often in situations where relationships may be possible, and the reality of that becomes more evident to them;

“*When I did have a girlfriend, her mum and dad knew me well and if I had to go through that again… if a family asked me if I had a criminal record and stuff … I would have to be honest, and then it would be up to the family what they decide.” [low, 075]*

As with the approach adopted toward friendships among those who reside in the community, intimate relationships also appear to be treated with caution;

“*if I get too involved or committed with someone, and then they find out about me,...it's sort of bad blood [pause] you didn't tell me this and you know…[trailed off speech].” [Community 154]*

#### Openness

Honesty as the best policy was adopted by individuals resident within high or medium secure FMHS;

“*I tell the truth about my past and don't hide it.” [high/medium 130]*

Although friendship was a strong theme among individuals residing within low-security FMHS or open wards, there is also some evidence of carefully considering how open to be and the potential consequences of giving too much of themselves away;

“*Sometimes I think you could tell someone something about yourself, and then they can use it against you when you are ill.” [low 003]*

The protectionist attitude is again evident among those living in the community;

“*I don't tell lies about myself. I sometimes miss out things that perhaps I should mention. I pick and choose what to say.” [Community 079]*

#### Community

When asked about being part of a community for those living in a high or medium secure FMHS, the community was generally considered to be a place beyond the boundaries of the hospital rather than a group of people living together locally in their social environment:

“*[on transfer] well, I'll be a step nearer to the community.” [high/medium 159]*

The notion of a hospital or ward community as a social environment appeared more prevalent to those resident in low-security FMHS or open wards;

“*We have community meetings in here, and we bring up things that we would like to do.” [low 074]*

Individuals living in the community at 20 year follow-up have achieved the long-desired goals of independence and personal freedom. However, they also reported self-imposed limitations in their lives, such as purposefully frequenting busy areas to maintain anonymity:

“*Basically, what I do is leave the flat, get the bus out of the area, I do what I have to do in the town, get my shopping done up there, get the bus and go back into my flat.” [Community 154]*

## Discussion

This study examined the outcomes of a cohort of patients who were initially explored as part of a whole population survey and followed up 20 years later within the context of recovery. The study explored a range of outcomes and attitudes across different security levels, encompassing various aspects of clinical, functional, offender, social, and personal recovery. Recovery is a complex and multidimensional concept, and while the different aspects of recovery are reported individually in this study, they are interrelated rather than mutually exclusive.

The recovery aspects explored must be interpreted within the context of the high rates of premature deaths observed among this cohort, which are pervasive among adult psychiatric populations (28, 29). While the distinct lack of traumatic and suicide deaths is reassuring and markedly different from similar study cohorts (30), the high rate of natural deaths remains a cause for concern (19). Schizophrenia was the primary diagnosis for 70% of the cohort, making antipsychotic medication an essential tool widely used for reducing symptoms and easing distress. Respiratory disease/cancer was the primary cause of death, and although undoubtedly the legacy of an era when smoking was commonplace, smoking remains a problem for forensic patients after discharge from controlled inpatient environments. Morbidity analysis revealed no significant differences in cardiovascular, metabolic disease diagnoses, side effects associated with the use of antipsychotic medication (31) between those who died prematurely and those who survived. This suggests that antipsychotic medication was not a significant contributing factor. While it is important to holistically explore mental health recovery, the burden of physical disease and external causes of injury among this cohort should not be overlooked. Emphasizing the synergy between good physical and mental health is essential to promoting overall recovery.

### Clinical recovery

An examination of the journey through services demonstrates that the road to recovery from high secure care is often anything but a straight path. Within this cohort, the average post-baseline admission to high-secure care was almost 7 years, with those subject to restrictions on discharge admitted for just over 9 years. On average, individuals experienced four admissions/transfers to an alternative security level (the baseline admission is noted as 1). The admission length we observed may be partly due to the relative lack of medium secure services in Scotland at the time the original cohort was gathered. The first medium secure unit opened in 2000, with another two units opening in 2007 and 2013. Where full journey data were available, 45.3% experienced at least one readmission to a higher level of security following progression to a lower level/community, with 42.7% moving downwards through security levels without any readmissions. In a comparison of English general and forensic patients based in the community, Hodgins et al. (32) noted that FMHS adopted a lower threshold for readmission in terms of symptoms and medication non-compliance than general services. This may account for almost a quarter of the cohort progressing to community residence but continuing to experience occasional admissions. We observed that 12% of the patients remained in high secure care from baseline to December 2014 or date of death. This likely reflects the need for continued care within a high secure environment to appropriately manage risk to the general public. While this rate is roughly half that of long-stay patients (10 years of continuous high-security care) within an English sample (33), unlike English forensic mental health services, Scottish forensic establishments do not generally admit individuals with a primary diagnosis of personality disorder. Such individuals may require a longer admission for levels of risk to the public to be suitably managed. Including location at point of death, slightly less than a third transitioned to community living, highlighting that, for some individuals, achieving the goal of community living may be unrealistic. Instead, creating a sense of home, empowerment, and a life with meaning and purpose within a secure environment that provides optimal choices may be more appropriate recovery goals (34, 35). Indeed, there is a recognized need for long-term inpatient rehabilitation services to support forensic patients who are diverted to general adult services (36). Unlike Völlm et al. (37), who reported high rates of personality pathology in long-stay high or medium secure patients, ASPD was not associated with having remained within high or medium secure services during follow-up. However, it should be noted that, in Scotland, offenders with a primary personality disorder diagnosis generally remain in the criminal justice system, which is reflected in only 5% having a primary ASPD diagnosis. However, in line with Duke et al. (33), we noted that individuals subject to restrictions on discharge were most likely to remain within high or medium secure services, as were those who had experienced a higher number of years in high secure care prior to baseline study and who scored higher on the depression component of the Manchester Rating Scale (24) at baseline. Resnick et al. (38) highlighted that the four identified recovery domains, satisfaction with life, hope, empowerment, and knowledge of the mental illness, were strongly associated with lower severity of depressive symptoms. It has been suggested that greater management of subjective and objective depressive symptoms may reduce disengagement and enhance engagement in recovery-oriented tasks. Depression has also been reported as being associated with relapse among those with a schizophrenia diagnosis (39) and in relation to completed suicide, where depression is considered a more significant factor than command hallucinations (40).

### Functional recovery

In existing studies, functional recovery is generally associated with a return to premorbid levels of functioning in relation to the diversity of skills required for daily living, which can encompass those required for independent or supported living, employment and training, and developing and maintaining relationships (17). More recently, attempts have been made to define and operationalise functional recovery among individuals experiencing schizophrenia; however, no standardized definition could be established despite a majority of clinicians displaying a common understanding of what they considered functional recovery to be (41).

Within our cohort and specifically in relation to social roles, the overwhelming majority of cohort members described themselves as single (85.5%) at baseline, with only around a quarter (23.2%) indicating that they had ever been employed, and these findings were similar to those of Völlm et al. (37). These rates had not improved by follow-up, by no reviewed individuals in paid employment and three-quarters of deceased individuals noted as single. Although engagement with occupational therapy (OT) services did significantly improve from baseline to follow-up among those remaining as inpatients, and there was some engagement with supported work/project placements, similarly impaired functional features have been reported among French forensic parricidal patients followed for 15 years (42).

At baseline, this cohort represented the general sociodemographic characteristics of other forensic cohorts in terms of raised childhood adversities (43) and history of substance misuse (44). However, the low prevalence of primary and comorbid personality disorders indicates that, after a resolution of psychotic symptoms, fewer individuals should be left with damaged personalities, which may impact their social and functional recovery (37). Therefore, with appropriate levels of support, good recovery levels should be attainable.

Further functional recovery data at baseline and 20 year follow-up were obtained through ward/community keyworker interviews (20). When explored by lowest security level groupings, differences were observed in the baseline scores in terms of overall dysfunction and disturbed ward behavior, with those remaining within high or medium secure care evidencing higher median ratings than those progressing to residence within the community. However, no significant difference was observed in terms of nurses' opinions of dysfunction or dysfunction evident in OT-related activities. This suggests that more global aspects of functioning, rather than a prediction of how a patient may be able to cope in given situations or how they manage specific tasks, are more predictive of long-term recovery within this cohort. Where performance over time for specific individuals could be explored, those who remained in high or medium secure care displayed significant increases in nurse-rated dysfunction and dysfunction in relation to contact with the outside world, suggesting that these individuals were experiencing deteriorating functioning (45), while those progressing to low/open wards exhibited a significant decrease in disturbed ward behavior suggestive of stable-good functioning (45).

Roosenschoon et al. (46) demonstrated that coping, in terms of behavioral or cognitive efforts to manage situations that are appraised as stressful (47), was more relevant to the degree of functional and personal recovery attained than the level of clinical recovery achieved and that social support, while not associated with clinical recovery, was weakly associated with functional recovery. As in social recovery, self-stigma is also detrimentally associated with functional recovery, with a greater influence than social stigma (41). In this follow-up, there was a focus on societal markers of functional recovery, for example, in terms of paid employment and relationship status. Forensic patients who are doubly impacted by social stigma in relation to mental health and previous offending behavior may be better supported through the development of holistic coping strategies and a reduction in self-stigma. Individual functional and social recovery goals that are pro-social and health-affirming should be encouraged (48) to support the development of a life with meaning and purpose for individuals.

### Offender recovery

Offender recovery is considerably intertwined with personal recovery in terms of the renegotiation of identity (35, 49, 50). Central to the development of offender recovery is subjectively acknowledging and accepting the offending behavior, personal qualities and motivations involved, and the personal and social impact of their offense. This recovery task involves recognizing and integrating offending aspects with other often conflicting and competing identities, e.g., trauma survivor, person with severe mental illnesses, and the development of a sense of self distinct from that of an offender (17). Future offending may not be avoided without appropriately addressing underlying aspects of offender recovery (17).

We acknowledge that, in line with other recidivism research among mentally disordered offenders (MDO) (29, 51), the subjective exploration of offender recovery was overlooked in favor of more easily attainable and objectively measurable offenses committed, which, as Fazel et al. (29) have noted, are the most readily available outcome indicator within the literature. There were 54 recorded convictions for 20 individuals, representing an overall conviction rate of 22.7% for consenting/deceased traceable cohort members. Notably, no offense resulted in a loss of life, and there were no contact sexual offenses. Among this cohort, previous offending history or source of admission (court/prison/hospital) was not associated with post-baseline offending. These results are similar to those of another forensic cohort, with overall conviction rates of 27% (52), while our rate of violent recidivism (7.9%) is lower than the reported 20% (52). Our overall offending rates were lower than those reported through a meta-analysis of recidivism among MDOs, with unweighted overall offenses at 39% and violent offenses at 23% (53).

This cohort was diagnostically diverse, with only 5% experiencing a primary diagnosis of antisocial personality disorder (ASPD) and 29% comorbid ASPD at baseline (19), reflecting the position within Scottish FMHS that individuals with a primary diagnosis of personality disorder remain within the criminal justice system. The relatively low rate of violent reoffending observed may be reflective of the low numbers of antisocial personality patterns and the lack of association between previous criminal history, source of admission, and overall recidivism (54). The general lack of social and relational functional recovery observed, which reflects moderate risk factors, particularly among residents within the community where opportunities for recidivism are greatest, may be supportive of general reoffending behaviors. Our findings are also supported by outcome research asserting that treatment within FMHS leads to lower recidivism (55, 56) and, indeed, is more successful than general adult services in preventing aggressive behavior post-discharge, as general services do not examine previous antisocial/aggressive behavior (32).

### Personal recovery

The QPR is a quantitative tool developed primarily to examine recovery from psychosis (21). The QPR operationalises recovery as a process and maps onto the empirically derived categories of the CHIME framework of personal recovery (57): connectedness, hope and optimism about the future, identity, meaning in life and empowerment. As a tool, the QPR also exhibits significant inverse relationships between recovery from symptoms of psychosis and general psychological symptoms (21). This supports empirical findings from Resnick et al. (38), suggesting that depressive symptoms may be significantly and negatively related to recovery, e.g., feelings of hopelessness will impact a sense of recovery, as hope is considered an essential foundation of recovery (50, 58).

We examined QPR scores by security levels at the point of interview and, as expected, noted a significant difference in recovery between those who were detained within high-secure care and those who had progressed to low/open wards. The fact that there was no significant difference between individuals remaining in high or medium secure care and those currently living within the community was more unexpected and may be accounted for by the apparent lack of social recovery and self-protective measures adopted by those now residents within the community environment. Through exploration of clinical recovery, we noted that remaining within high or medium secure care throughout the 20 year follow-up was associated with sub-score for depression (the Manchester Rating Scale, 19) exhibited at baseline (1992/93). Within this cohort, the dominant diagnostic group was schizophrenia, and it has been reported (59) that rates of depression among individuals with schizophrenia vary with the stage of illness, from estimates of up to 60% while in acute episodes to 20% during chronic phases and 50% after treatment of the first episode. Persistent clinical/subclinical depressive symptoms may impact the establishment of recovery processes. They may also be related to the negative symptoms of schizophrenia, which are associated with poorer outcomes (60).

Within the literature, affective symptoms at onset are associated with better outcomes in first-admission psychosis (61). It should, however, be noted that the majority of members of this cohort were not necessarily experiencing a first episode of psychosis. At baseline (1992/93), the mean lifetime psychiatric stay was 9.3 years (range 0.08–45 years), indicating the chronic nature of psychotic illness that is a characteristic of many patients located within forensic psychiatric services (62).

While exploring the potential relationship between depressive symptoms and recovery, we examined the more nuanced scores obtained through the MADRS (63). We were able to identify that subjective sadness at baseline (1992/3), first follow-up (2000/01) and 20 years of follow-up (post-2014) were all negatively correlated with QPR recovery scores at the 20 year follow-up, indicating that internalized feelings of sadness, not necessarily objectively discernible markers, may impact the foundation of hope and the process of recovery.

FMHS have embraced person-centered recovery-based philosophies that are rooted in the subjective experience and conceptualized as an attitude or orientation (38). Therefore, fostering personal recovery in FMHS must balance the goal of supporting personal autonomy with the organizational need to manage risk (64). To address risk, FMHS must strive to offer optimal recovery choices that foster the development of hope and are tailored to the personal needs of their patients (35).

Analysis of qualitative data from our cohort of individuals who are now residents in the community reflecting upon their journey from the high secure state hospital to their current community placement yielded a picture of progress and personal development. The findings have been interpreted in terms of the three primary themes of personal forensic recovery (safety and security, negotiating changing identities, and the development of hope), highlighted through meta-syntheses by Clarke et al. (35) and Shepherd et al. (50).

It has been suggested that for recovery to develop within the individual, they must feel a sense of safety and security (49, 50). The source of this sensation may arise from the physical environment and/or associated restrictions. This combination may create a space that facilitates personal growth and development. Within this cohort, who were somewhat removed from their experience of The State Hospital by many years, discussion and description of the many barriers to recovery painted their early experiences of the environment as less than safe. This both supports and is in contrast to the findings from Laithwaite and Gumley (49), from the same hospital, who reported admission as providing individuals “respite” from their experiences but also acknowledged that others found the whole experience frightening. These barriers to recovery were generally diminished over time as the main drivers of recovery; medication and psychological therapies provided a strong foundation, and individuals began to experience respite from intrusive symptoms through medication compliance and the development of appropriate coping mechanisms.

Negotiating changing identities is reported as another major theme (35, 49, 50), with individuals beginning to recognized and integrate different self-identities, e.g., an addict, an offender, a person with severe mental illness and any legal labels associated with that, e.g., insane, and the development of a new identity. This cohort provided evidence of individuals' changing identities, with several adopting the identity of ward troublemaker in the early days of their admission to display their frustrations. However, through identity work to develop self-understanding, acceptance, and the enhancement of self-esteem, individuals spoke of maturing, turning their attention to recovery, and moving on. Self-stigma and social discrimination in relation to living with a severe mental disorder, particularly schizophrenia (65), can prevent acceptance of a mental ill health identity and impact the promotion of self-esteem (66) and the process of personal recovery.

As mentioned, the development of hope is also considered essential to the establishment of the process of recovery (50). Again, it may be the result of the passage of time, but there was little discussion of “hope” as a concept from which recovery took root in these interviews. There was mention of the hopelessness of their situation within the high secure environment stemming from admission and institutional battles relating to delayed discharge pathways. Among individuals who presented within the community with a more fragile sense of recovery and mental health stability, hope and recovery were more active concepts. Shepherd et al. (50) and Laithwaite and Gumley (49) also note that supportive relationships are important in conveying to individuals how positive self-change can be viewed by others. Individuals spoke of the importance of formal and informal feedback from staff and its impact on them. As noted by Laithwaite and Gumley (30), the concept of staff trust was also important in shaping behavior.

### Social recovery

Where individuals are obliged to undertake a programme of care, FMHS also have a duty of reciprocity, to provide safe and appropriate services to foster and support the development of rehabilitation and recovery. Recovery should provide the opportunity for individuals to become empowered, exert control, and create lives beyond the confines of their illness that reflect their life goals at both personal and social levels (2). One of the most striking recovery features of this cohort was the lack of social recovery observed, particularly among those living in high or medium levels of security and individuals who had negotiated a pathway to residence in the community. This study provided a unique opportunity to qualitatively explore social recovery across security levels.

Individuals' sense of connectedness (35), as demonstrated through reflection on the importance of friendships and family, demonstrated a disparity between family and peer relationships within higher levels of security. Family relationships were presented as being far more important than establishing peer and staff relationships, with the notion of community as a social unit completely discounted. Overall, these individuals had remained in high or medium secure services since the baseline study (1992/93), reflecting greater illness or offense severity and a reduced ability to engage in or progress through recovery tasks.

As reflected in this cohort (14), forensic patients often experience impoverished and chaotic social conditions (67) and a high number of adverse life events (8). Such early life experiences of disrupted or malignant relationships can lead to secure hospital admission being interpreted as either a place of safety or a continuation of a negative environment, leading to relationship distrust (49) and the creation of barriers such as a “them and us” perspective (68). The lack of development of staff/peer relationships and a sense of social community among our high or medium secure cohort points toward continuing relationship distrust and, subsequently, a delay in refining and developing their sense of self (49), resulting in stunted personal growth and recovery. However, the reported importance of family could be construed as evidence of the successful development or repair of family relationships. These findings, reflecting the importance of contact with family and/or others from outside the secure environment, echo those reported by Völlm et al. (37), who also noted that clinical experience suggests that it is the re-establishment of family relationships rather than maintenance that accounts for contact. It is, therefore, possible that within our findings, family relationships may be idealized.

In contrast, individuals resident in low/open wards demonstrated an understanding and appreciation of the importance of family, staff/peer relationships, and the ward social community. This was associated with a deeper ability to generally engage with their recovery tasks, as evidenced by their progression beyond high secure care. Greater access to the community and vocational projects commensurate with location within low/open wards can ameliorate barriers to social recovery created by detention in high or medium secure services (69). Hospital residence with reduced security offered a safe environment in terms of social and professional support through which to practice social skills, develop employment skills, and gain exposure to and engagement with the community environment.

Those who had transitioned to life in the community reverted to superficial and isolative behavior from a position of general social engagement and confidence, which was in contrast to that observed among those residing in low/open wards. This change in perspective appears to be internally driven, with community-dwelling individuals acknowledging engagement in casual social relations but expressing little desire to establish deeper interactions. This self-protectionist stance is also reflected in community residents' approaches to intimate relationships, general openness, and a sense of community. Although they have achieved long-sought independence and freedom that comes with moving out of the hospital environment, community residents demonstrated self-restricted social lives.

## Conclusion

This overview of the clinical, functional, offender, social, and personal recovery of a cohort of 241 individuals cared for in high secure forensic care during 1992/93 has highlighted a range of issues; 36.9% of the cohort died at an average age of 55.6 years, and FMHS must do more to support and encourage the physical wellbeing of patients to ensure that individuals live long, healthy lives and can enjoy the improvements in mental health that the majority develop. Over 40% progressed through secure care without any readmissions to higher levels, and only 12% could not move on from high secure care. Recidivism rates were very low. Although symptom management was very good, empirical measurement of recovery for schizophrenia provided a poor result. This, however, needs to be interpreted alongside personal recovery, which evidenced good insight, self-development, and an overall positive reflection upon their recovery journey. It was noted that subjective sadness/depressive experiences might impact not only personal recovery but more widely across the recovery process. Social and functional recovery, as interpreted through societal measures, was not great, with individuals failing to develop intimate relationships or secure open employment and fearing others finding out their past, leading to rejection or persecution. Although there is some evidence of self-stigma which can be more impactful upon recovery than societal stigma, mental health stigma within society remains pervasive. Until more is successfully done to address the issues, many forensic patients will restrict their social engagement.

## Limitations

There are several limitations associated with this study. We were unable to secure cross-border communication with England and Northern Ireland to obtain gatekeeper information for cohort members residing within their regions. Similarly, we could not request information for those residents overseas. This restricted the cohort to those remaining resident within Scotland, with the exception of the mortality status and death certificate information relating to those resident within England/Wales. As a gatekeeper approach was adopted, the invitation to participate could only be made to those for whom a suitable gatekeeper could be located and where the gatekeeper advised that the cohort member held the capacity to consent and remained mentally and physically well enough to participate. Although permission had been sought for the health records of deceased members to be reviewed, the length of follow-up and the year of death meant that some volumes had been destroyed.

Similarly, a small number of volumes were not available for all living participants. Health information could only be requested for deceased and consented participants. Not all deceased/consented participants could be traced by Police Scotland, and some offenses may have been weeded from the Criminal History System in accordance with their Weeding and Retention policy (26). Criminal data/any police contact was also noted through the case note review, and it is therefore unlikely that any serious offending was missed. Convictions relating to those lacking capacity or too unwell to participate, study refusers, residents of England/Northern Ireland, and deceased individuals removed from the dataset by Police Scotland have been omitted from the analysis. Functional recovery data at baseline and the 20 year follow-up were sourced from ward/community keyworkers only rather than collated from the recommended three primary sources: patients, relatives or caregivers, and the clinician (41).

## Data availability statement

The raw data supporting the conclusions of this article will be made available by the authors, without undue reservation.

## Ethics statement

The study involving human participants were reviewed and approved by South East Scotland Regional Ethical Committee 01, reference 15/SS/0015. The patients/participants provided their written informed consent to participate in this study.

## Author contributions

Conceptualization and writing—review and editing: LT. Design and methodology: LT and CR. Conduction of the study, statistical analysis, interpretation, and writing—original draft preparation: CR. All authors contributed to the article and approved the submitted version.

## Footnotes

^1^One person was double counted as they died following participation.
