# Supplementary material for: Long-term outcomes of the recovery approach in a high-security mental health setting: a 20 year follow-up study
Source: Front Psychiatry. 2023 May 11;14:1111377. doi: 10.3389/fpsyt.2023.1111377 (PMC10213922; doi:10.3389/fpsyt.2023.1111377)
Supplement: Supplementary file 1 [file Data_Sheet_1.pdf]

Supplementary material

Appendix A.

Tools applied during participant interview

**Tools applied during participant interview**	**Baseline (1992/93)**	**first follow up (2000/01)**	**20+ year follow up (2015 onwards)**	**20+ year follow up recovery aspect**
Standardised psychiatric assessment for chronic psychotic disorders (The Manchester) (Krawiecka *et al.* 1977)	x	x	x	Clinical
Mania Rating Scale (Bech *at al.,* 1978)	x			
Depression Rating Scale (MADRS) (Montgomery and Åsberg, 1979)	x	as subscale of CPRS	as subscale of CPRS	Clinical
Assessment of Involuntary Movements Scale (AIMS) (Guy, 1976)	x	x		
Scale for Targeting Abnormal Kinetic Effects (TAKE)(Wojcik *et al.,*1980)	x	x		
National Adult reading Test (Nelson, 1982)	x			
Diagnostic and attainment test (Schonell and Schonell, 1950)	x			
QUICK Test (Ammons and Ammons, 1962)	x	x	x	Functional
Comprehensive Psychopathological Rating Scale (CPRS) (Åsberg *et al.,* 1978)		x	x	Clinical
Rating Scale for drug induced akathisia (BARNES) (Barnes., 1989)		x		

Brief Psychiatric Rating Scale (BPRS) (Overall & Gorham, 1962)		x		
Scale for the assessment of negative symptoms (SANS) (Andreasen, 1984)		x		
Social Dysfunction and Aggression Scale (SDAS) (Wistedt *et al.,* 1990)		x	x	Clinical/ functional
Questionnaire about Process of Recovery (QPR) (Neil *et al.,* 2009)			x	Personal/social
Semi structured interview based on 7 elements of recovery.			x	Personal/social

Appendix B.

Tools applied/data collected through case note review

Mental health case notes from 1992/3 – 2014 were located using pathway information supplied by NSS. Each service was visited and where appropriate notes retrieved from archive to be reviewed on site (local mental health hospital). For a very small number of deceased individuals case records were collected from off-site storage facilities and brought into The State Hospital Health Records department for review.

**Information obtained by case note review**	**Baseline (1992/93)**	**first follow up (2000/01)**	**20+ year follow up (2015 onwards)**	**20+ year follow up recovery aspect**
St Louis criteria (Feighner, *et al.*, 1972)	x			
PSE Syndrome checklist (Wing *et al.*, 1967)	x			
Demographic details	x	x	x	social
Legal status	x	x	x	offender
Psychiatric history	x	x	x	clinical
Drug history	x	x	x	Clinical/ functional
Medical and forensic history	x	x	x	Clinical/ functional/ offender
Admission details	x			
Social and personal history	x	x	x	Social/ functional
Family history	x			
Diagnoses	x	x	x	clinical
Clinical features	x	x	x	clinical
Violence Risk Appraisal Guide (VRAG) (Harris *et al.*, 1993		x		
HCR - 20 (Webster *et al.*, 1997) Historical-10 applied.		x		
The Psychopathy Checklist - Revised (PCL-R) (Hare, 1991)		x		

Appendix C.

Tools applied/data obtained from mental health staff and Police.

**Information obtained by staff interview**	**Baseline (1992/93)**	**first follow up (2000/01)**	**20+ year follow up (2015 onwards)**	**20+ year follow up recovery aspect**
Social Dysfunction and Aggression Scale (SDAS) (Wistedt *et al.,* 1990)	x			Clinical/ functional
Disability Assessment Schedule (DAS) (Jablensky *et al.*,1988)	x			Functional
**Information obtained from Police Service**				
Summary of criminal history 1992-2015				Offender

Appendix D.

Participant interview schedule (quantitative)

All potential participants had the study introduced to them by the single researcher within their ward environment for those who remained within inpatient services. For those who were resident within the community the researcher was accompanied by the CPN of the individual of interest to their usual appointment location which was either within an outpatient service or within the individual's own home. They were introduced by the ward staff/CPN to the individual and then made the study introduction. Interviews were generally conducted on a second visit and on a one to one basis.

Individuals who consented to study were offered the opportunity to opt out of having the interview recorded. All of those consenting to study agreed to audio recording.

To accommodate the needs of individuals the interview was split into sections as required and repeat visits made if needed to complete the quantitative and qualitative schedules.

Section 1.

Comprehensive Psychopathological Rating Scale (CPRS) (Åsberg *et al.,* 1978)

Items 1-40 reported by participant, rated by researcher

Items 41-65 observed by researcher, rated by researcher

Items 66 Global rating of illness, rated by researcher

Items 67 Assumed reliability of the rating, rated by researcher

Social Dysfunction and Aggression Scale (SDAS) (Wistedt *et al.,* 1990)

Items 1-9 reported by participant, rated by researcher

Item 10 self-harm, reported by participant, rated by researcher

Section 2.

General questions on aggressive/antisocial incidents

In the last month have you been involved in any capacity, in any incidents that have led to police involvement.

Yes - *complete table below*

No – *move onto next question*

Type of Behaviour	Date	Location	Charged?	Convicted?	Disposal	Influence Drug/Alcohol

			Yes/No	Yes/No		Yes/No

In the last month have you been involved in any capacity, in any other incidents involving verbal or physical aggression or violence?

Yes - *complete table below*

No – *move onto next question*

Type of Behaviour	Date	Location	Consequence	Influence<br>Drug/Alcohol
				Yes/No

<u>Medication</u>

Are you currently prescribed any regular medications? **Yes/No** *(if no move onto next question*

What medications are you prescribed? *(Complete table below)*

Are there any medications that you do not take as prescribed? *(Complete table below)*

What are the reasons for not taking those medicines?

medication	dose	route	compliant	Reasons for non-compliance
			Yes/No	

<u>Alcohol and substance use</u>

Have you been drinking alcohol over the last month? **Yes/No** *(if no move onto next question)*

How do you feel when you stop drinking? (ask questions exploring withdrawal/ dependence symptoms)

Number of drinking days	What drank, how much (*to allow unit calculation. E.g. 2L frosty Jacks cider)*	Withdrawal symptoms	Dependency symptoms

Have you taken any non-prescribed drugs in the last month? **Yes/No** *(if no move onto next question)*

Type	Number of days using	Doses per day	Route(s)	Withdrawal symptoms	Dependency symptoms

<u>Contact with services</u>

How often do you see your keyworker?

1	Daily
2	Couple of times a week
3	Weekly
4	Fortnightly
5	Monthly
6	Intermittently
7	Never

How often do you see a psychiatrist?

1	Daily
2	Couple of times a week
3	Weekly
4	Fortnightly
5	Monthly
6	Intermittently
7	Never

Section 3.

QUICK IQ Test (Ammons and Ammons, 1962)

Questionnaire about Process of Recovery (QPR) (Neil *et al.,* 2009)

Appendix E.

Semi structured interview based on 7 elements of personal recovery (additional probing as required).

Recovering from an illness or finding ways to change the way you behave is a very personal experience. Although there are many similarities, no two people recover in the same way.The journey towards recovery can mean different things to different people.

I would like you to think about the difficulties that brought you to the State Hospital to be cared for at some point during 1992/93. With that in mind I would like to ask you,

Questions

What does recovery mean to you?

Where on your recovery journey do you see yourself?

What has been most helpful in your recovery?

What has not been very helpful?

Looking back over the last 20 years since we first spoke to you, have there been any memorable parts of your journey that made you change direction?

*If **Yes**: Do you mind telling me a bit about them?*

*If **No:** Do you feel that you have changed at all since we first spoke to you 20 years ago? In what way? **OR** Why do you think you have found it hard to change?*

<u>Hope</u>

Do you believe that you can recover?

*If **Yes:** When did you first really believe you could recover?*

What helps you hold onto that belief when you are finding things difficult?

*If **No:** Have you ever believed you could recover?*

Yes *What happened to make you feel that you couldn't get better?*

What do you think would help you to find that belief again?

OR

No *What do you think would need to happen to make you believe you could get better?*

<u>Secure Base</u>

What do you think of the place where you live?

If Positive*: do you think it has been helpful in your recovery? Explore.*

If Negative*: do you think it been unhelpful in your recovery journey? Explore.*

(If applicable) *What do you think about the staff? Explore.*

Sense of self

It has been 20 or so years since you were first interviewed in the State Hospital…

Has the way you see yourself changed over that time? *Explore.*

What has been the impact of that (change/lack of change) on your recovery journey? *Explore.*

Supportive relationships

(*should know a bit about relationships from the CPRS etc)*

Have you been able to develop friendships where you can be yourself *(not hide your past illness/difficulties)?*

Can you tell me a bit about them?

Do you have any other people (*family or professionals)* who give you support?

Do you find yourself having to tell lies about your past? *(Explore - to avoid stigma, fill in blanks in history…)*

Empowerment and Inclusion

Can you make decisions about your recovery?

Does someone else make any decisions?

How does this make you feel?

Do you feel part of a community?

Do you feel part of the wider world?

Coping strategies

Have found the right skills to help you manage your illness?

What works the best for you?

Do you still find yourself using strategies that you know aren't good for you in order to cope? *Explore (self-harm, destructive, aggressive etc.)*

Meaning and purpose

What sorts of things make you feel happy with life?

What sorts of things do you do that make you feel like you have achieved something? (work/placement activities/sports other activities)

If ***Negative****: What do you think would allow you to feel happy?*

Post interview the researcher rates observed items of CPRS and completes the Standardised Psychiatric Assessment for Chronic Psychotic Disorders (The Manchester) (Krawiecka *et al.* 1977)

References for supplementary material

Ammons R.B. & Ammons C.H. (1962). *Quick Test*. Missoula, MT: Psychological Test Specialists.

Andreasen, N.C. (1989). Scale for the Assessment of Negative Symptoms (SANS). *The British Journal of Psychiatry*, 155 (Suppl. 7) 53-58.

Asberg, M., Perris, C., Schalling, D., & Sedvall, G. (1978). The Comprehensive Psychopathological Rating Scale (CPRS) - Development and Application of a Psychiatric Rating Scale. *Acta Psychiatrica Scandinavica*, Suppl. 271.

Barnes, T.R. (1989) A rating scale for drug induced akathisia. *British Journal of Psychiatry*, 154: 672-676.

Bech, P., Rafaelsen, O.J., Bolwig, T.G. & Kramp, P. (1978). The Mania Rating Scale: scale construction and inter-observeragreement. *Neuoropharmacology,* 17 (6):430-1.

Feighner JP, Robins E, Guze SB, Woodruff RA Jr, Winokur G, Munoz R. Diagnostic Criteria for Use in Psychiatric Research. *Arch Gen Psychiatry*. 1972; **26**(1): 57- 63.

Guy, W. (1976) *ECDEU Assessment Manual for Psychopharmacology*: Revised (DHEW publication number ADM 76-338). Rockville, MD, US Department of Health, Education and Welfare, Public Health Service, Alcohol, Drug Abuse and Mental Health Administration, NIMH Psychopharmacology Research Branch, Division of Extramural Research Programs, 534-7.

Hare R. *The Hare Psychopathy Checklist–Revised*. Toronto, Canada: Multi-Health Systems; 1991.

Harris GT, Rice ME, Quinsey VL. Violent recidivism of mentally disordered offenders: The development of a statistical prediction instrument. *Crim Justice Behav*. 1993; **20**: 315- 335.

Krawiecka, M., Goldberg, D., & Vaughan, M. (1977). A standardized psychiatric assessment scale for rating chronic psychotic patients. *Acta Psychiatrica Scandinavica*, 55: 299–308.

Montgomery, S.A., & Asberg, M. (1979). A new depression scale designed to be sensitive to change. *British Journal of Psychiatry* 134 (4): 382–89

Neil, S., Kilbride, M., Pitt, L., Nothard, S., Welford, M., Sellwood, W., & Morrison, T. (2009). The questionnaire about the process of recovery (QPR): A measurement tool developed in collaboration with service users. *Psychosis*, 1(2):145-55.DOI: 10.1080/17522430902913450

Nelson, H.E. (1982) *National Adult Reading Test (NART): Test Manual.* Windsor: NFER-NELSON

Overall, J.E. & Gorham, D.R. (1962) The Brief Psychiatric Rating Scale. *Psychological Reports*, 10, 799-812.

Schonell, F.J. & Schonell, E.F. (1950) *Diagnostic and Attainment Testing* Oliver and Boyd, Edinburgh.

Webster CD, Douglas KS, Eaves D, et al. *HCR-20. Assessing Risk for Violence, Version 2*. Mental Health, Law and Policy Institute, Simon Fraser University; 1997.

Wing JK, Cooper JE, Sartorius N. *The Measurement and Classification of Psychiatric Symptoms: an Instruction for the PSE and CATEGO Program*. London: Cambridge University Press; 1974.

Wisedt, B., Rassmussan, A., Pederson, L., Malm, U., Träskman-bendz, L., Wakelin, J. & Bech, P. (1990). The Development of an Observer Scale for Measuring Social Dysfunction and Aggression (SDAS) *Pharmacopsychiatry,* 23 (6): 249-252.

Wojcik, J.D., Gelenberg, A.J., LaBrie, R.A. & Mieske, M. (1980) Prevalence of tardive dyskinesia in an outpatient population. *Comprehensive Psychiatry*, 21 (5) 370-80

World Health Organisation. (1988). The WHO psychiatric Disability Assessment Schedule. World Health Organisation, Geneva.
